# Study protocol of a German multi-center, observer-blind, randomized, and actively controlled parallel-group trial comparing maintenance electroconvulsive therapy to treatment as usual for relapse prevention in clozapine resistant schizophrenia

**DOI:** 10.1186/s12888-025-06990-2

**Published:** 2025-05-26

**Authors:** Anton Deicher, Sebastian Karl, Marie-Luise Otte, Johannes Knabbe, Bernadette Wendel, Maria Gose, R. Christian Wolf, Alexander Sartorius

**Affiliations:** 1 Department of Psychiatry and Psychotherapy, Research Group Brain Stimulation, Central Institute of Mental Health, Medical Faculty Mannheim, Heidelberg University, J5, 68159 Mannheim, Germany; 2 Department of General Psychiatry, Center for Psychosocial Medicine, Heidelberg University Hospital, Voßstraße 4, 69115 Heidelberg, Germany; 3 Coordination Centre for Clinical Trials (KKS) Heidelberg, 69120 Heidelberg, Germany; 4 DZPG, German Center for Mental Health, Partner site Mannheim/Heidelberg/Ulm (ZIHUb), Germany

**Keywords:** Electroconvulsive therapy, ECT, Maintenance electroconvulsive therapy, mECT, Clozapine-resistant schizophrenia, CRS, Treatment resistance, Relapse prevention, Randomized controlled trial, Schizophrenia

## Abstract

**Background:**

Schizophrenia is one of the most severe and costly mental disorders in terms of human suffering and societal expenditure. About 15–30% of patients do not respond to all known antipsychotics, including clozapine, the current gold standard in these cases. Electroconvulsive therapy (ECT) is well-known to be highly effective in clozapine-treatment-resistant schizophrenia (CRS), and synergistic effects of clozapine and ECT have been demonstrated. However, relapse rates after successful courses of ECT are still very high, and evidence for maintenance ECT (mECT) in CRS is scarce at best.

**Methods:**

Here, we present the protocol of the MECT-RESIST trial, a German multi-center, observer-blind, randomized, and actively controlled parallel-group clinical trial. The scientific aim of the study is to test the hypothesis that mECT plus treatment as usual (TAU) (intervention group) is superior to TAU alone (control group) for relapse prevention in CRS. The primary endpoint is time to relapse. Secondary endpoints include the proportion of relapse-free patients, the global level of functioning and quality of life, depressive symptoms, overall symptoms of schizophrenia, concomitant catatonic symptoms, stress and self-stigmatization and cognitive performance. We aim at randomizing 84 patients between 18 and 65 years with a clinically diagnosed CRS and brief psychotic rating scale (BPRS) > 45, who responded to a series of ECT (BPRS < 70% of initial BPRS), to either recieve mECT + TAU or TAU over a period of 28 weeks followed by a follow-up of 12 months. The study will be performed between 2025 and 2028.

**Discussion:**

In this multi-center trial, we aim to examine the effectiveness of mECT in CRS patients who improved after a course of routine ECT. If mECT will lead to a longer time to relapse and/or to a higher proportion of relapse-free patients compared to those undergoing treatment as usual, this trial would have an enormous impact on therapeutic strategies for patients with CRS and would induce a profound change of current treatment guidelines, where ECT still ranks at the level of ultima ratio, despite accumulating evidence suggesting otherwise.

**Trial registration:**

ClincalTrials.gov NCT06456983, registered 7 Jun 2024. Deutsches Register Klinischer Studien DRKS00036886, registered 14 May 2025.

**Supplementary Information:**

The online version contains supplementary material available at 10.1186/s12888-025-06990-2.

## Background

Schizophrenia (SZ) is one of the most severe and costly mental disorders in terms of human suffering and societal expenditure [1–5]. Life-time prevalence of SZ is about 0.5–1.0%. Sustained recovery occurs in less than 14% of patients within the first five years following a psychotic episode. Longer-term outcomes may be marginally better: a large international 25-year follow-up study reported an additional 16% with late-phase recovery. Throughout Europe, less than 20% of people with SZ are employed [4, 6]. Years of potential life lost typically range between 10 and 20 years [7].

A major challenge in treating SZ is that about 15–30% do not respond to all known antipsychotics, including clozapine, which is currently regarded as the gold-standard for treatment-resistant SZ [8]. These patients are among the most severely affected, with a poor prognosis in terms of medical and social rehabilitation. For these severely ill patients with persistent positive symptoms there are no further evidence-based alternatives or augmentation strategies. Hence, recent Cochrane database reviews stated that the quality of existent studies is too poor to recommend any intervention additional to clozapine and that new, properly conducted, randomized controlled trials independent from the pharmaceutical industry need to be performed to help these most severely impaired patients [9, 10].

Although ECT was initially used to treat SZ and is recommended in national treatment guidelines and by the German Association for Psychiatry, Psychotherapy and Psychosomatics (DGPPN) [11, 12], it is still by far underused in the therapy of SZ in many countries, including Germany. This may be due to stigmatization but could also be a consequence of the development of antipsychotic medication, which however possesses its own inherent limitations and disadvantages. ECT is well known to be effective in both treatment resistant and clozapine resistant SZ [13–18], and synergistic effects of clozapine and ECT have been demonstrated [19–23]. However, relapse rates after successful courses of ECT are very high [13], and evidence for the use of maintenance ECT (mECT) in treatment resistant SZ [24] or CRS [25] is scarce at best. At present, there are only case reports and a limited number of retrospective studies demonstrating reduction of clinical severity of the illness and re-hospitalization with mECT [15, 25, 26].

## Methods/design

### Study objective and endpoints

The main purpose of the study is to show superiority of mECT plus treatment as usual (TAU) versus TAU only. Thus, all patients will be enrolled in an initial course of ECT (phase I) and must respond to this treatment before being randomized to the treatment groups (phase II).

The primary endpoint is the time to relapse for which relapse is defined as BPRS ≥ 20% higher than individual BPRS at start of phase II at any following study visit or any unscheduled readmission due to a worsening of psychiatric symptoms or any unscheduled visit with an BPRS ≥ 20% higher than individual BPRS at start of phase II.

The BPRS is evaluated by an unblinded rater at the site and a blinded central rater. Hospital readmission is decided by unblinded non-study related staff. The first relapse decision is used for analysis.

If the condition of a patient is worsening (assessed by unblinded staff), the BPRS assessment is videotaped for the evaluation by the blinded rater.

The time to relapse is the most important outcome parameter, when the effectiveness of interventions in patients with SZ is investigated. The BPRS is a widely used instrument in psychiatry for the assessment of psychotic symptoms and has been implemented in previous clinical trials and large-scale investigations [27]. The instrument has been internationally validated and proven to be sensitive to change.

The secondary objectives are to compare side effect profile and safety as well as psychopathological changes between mECT plus TAU versus TAU only.

The secondary endpoint for treatment efficacy is the proportion of relapse free patients at the end of phase II.

Further secondary endpoints are:

- BPRS [27]
- Global Assessment of Functioning (GAF) allowing a general [28] and Positive and Negative Symptom Scale (PANSS) allowing a differentiated view on specific symptom dimensions [29].
- Hamilton Depression Score (HAMD) [30] monitoring depression, since ECT and mECT have a well-known positive influence on mood.
- Northoff Catatonia Rating Scale (NCRS-dv) [31] assessing the full spectrum of catatonic symptoms.
- Abbreviated Quality of Life Enjoyment and Satisfaction Questionnaire (Q-LES-Q-18) [32].
- The Self-Stigma of Mental Illness Scale– Short Form (SSMIS-SF) [33] and the Stigma-Stress-Skala [34] are used for self-labeling and stigma.
- Mini Mental State Examination (MMSE) [35] and THINC-it [36–38] monitoring cognition.

All secondary endpoints listed above are analyzed at the end of phase II and at the end of follow-up.

Safety will be also determined by the frequency of adverse events from randomization up to the last follow-up visit.

### Study design

**Fig. 1:**
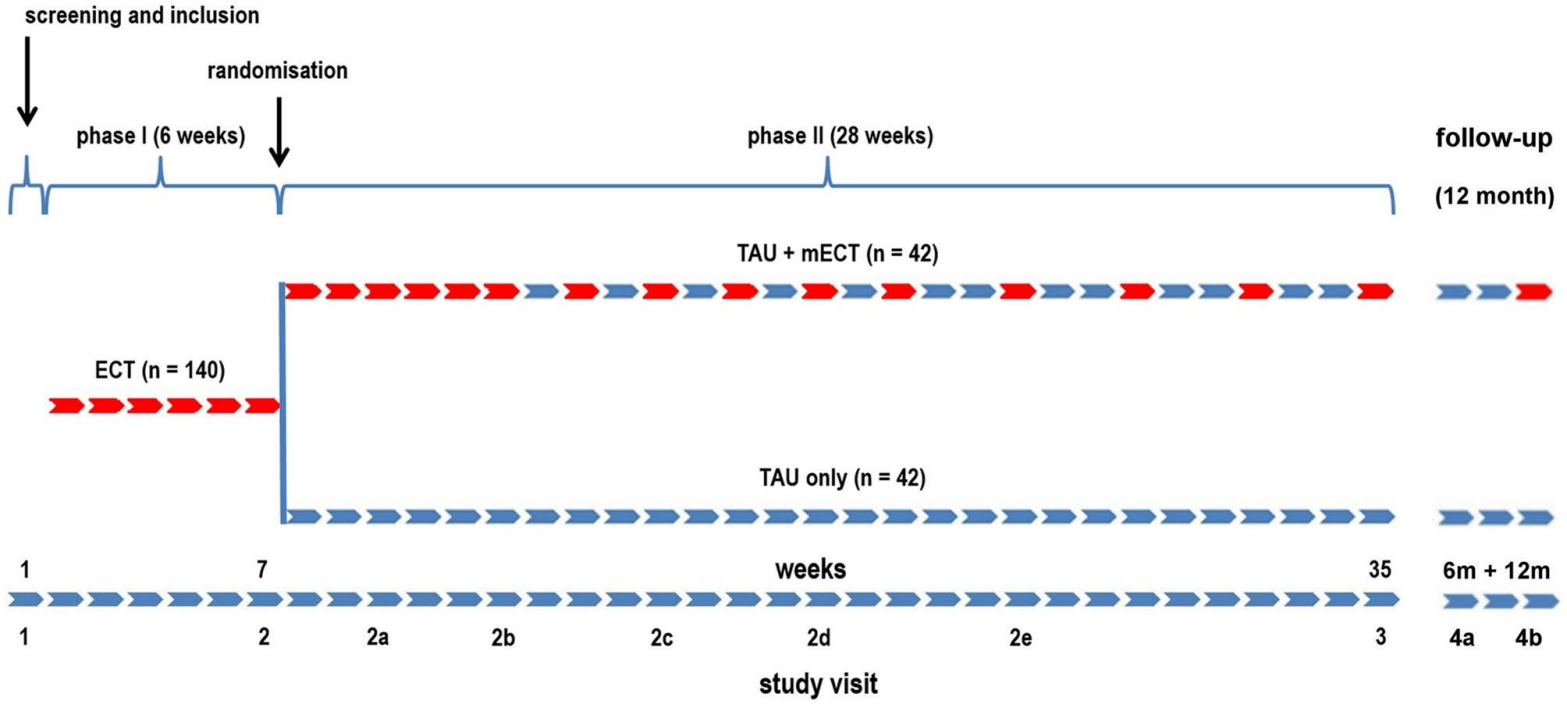
Schematic of the MECT-RESIST study design. Boxes describe weeks, with red boxes indicating weeks with ECT/mECTs

This is a German multi-center, observer-blind, randomized, and actively controlled parallel-group study to compare after a successful series of routine electroconvulsive therapy (ECT) the overall effectiveness of maintenance ECT (mECT) plus treatment as usual (TAU) versus TAU only in patients aged 18 to 65 years with the indication of clozapine resistant SZ. A schematic of the study design is shown in Fig. 1.

The study consists of two phases. In phase I, patients will be included in the study according to the inclusion and exclusion criteria (see section “Subject selection”) and will receive an ECT series with a maximum of 18 ECT treatments based on a clinical indication for ECT or until there is a clinically sufficient response to treatment. In phase II, patients who respond to ECT in phase I (defined as Brief Psychiatric Rating Scale (BPRS) score < 70% of BPRS at visit 1) meet the inclusion criteria for phase II of the study. After completing the ECT series in phase I and before starting phase II, patients are randomized to receive either mECT plus TAU or TAU alone. Patients will be randomized 1:1 by the site staff using REDCap (eCRF) [39, 40], which maintains allocation concealment by accepting patients’ inclusion before assigning the treatment. Block randomization will be applied stratified by patients taking clozapine at visit 2 and patients not taking clozapine at visit 2 but not site in order to prevent predictability of allocation. After the completion of phase II, there will be a 12-month follow-up period. In total, the duration of the study for each subject is expected to be at maximum 20 months.

The study will be conducted over a period of 54 months. The duration of the clinical phase will be 39 months. Recruitment started in February 2025 and is planned to continue through June 2028. The duration of the study may be extended and additional sites may be added depending on the observed rate of recruitment.

The interviews regarding BPRS will be videotaped at baseline (visit 2) as well as at any planned and/or unplanned visit at which a subject is experiencing a worsening of its condition. The videos will be edited to remove any hints about treatment, and brief (2–5 s), nonessential portions from all tapes will be erased to create 3–10 skips in a random fashion, thus balancing the presence of awkward skips that might be otherwise present mostly in the ECT tapes. An independent, blinded rater (psychologists and psychiatrists trained in the assessment of psychopathological symptomatology) will score the edited tapes.

The primary endpoint is a combined endpoint of judgements by blinded and unblinded non-study related raters. Strictly speaking, the study is only partially observer blind. Yet, blinding of the observer at the site is impossible because the patients usually reveal their actual treatment during assessments. Therefore, it is justifiable from a medical point of view to refer to this study as observer-blind.

### Subject selection

A total of 140 patients will be recruited in phase I of the study and 84 patients will be randomized (phase II), i.e. 42 patients per treatment group. The study will be national and multicenter. It is intended that the study will take place at approximately 14 German sites.

Patients meeting all of the following criteria will be considered for enrollment in the study:

- Current diagnosis of SZ according to Diagnostic and Statistical Manual of Mental Disorders, Fifth Edition (DSM-5).
- BPRS total score > 45.
- History of CRS, which will also include treatment-resistant SZ with clozapine intolerance or absolute contraindications for clozapine.
- Age between 18 and 65 years.

Patients presenting with any of the following criteria will not be included in the study:

- Diagnosis of DSM-5 major neurocognitive disorder (“dementia”), current severe substance-use disorder, affective disorders with psychotic symptoms or any personality disorder.
- Inability to read/write German or inability to provide written informed consent.
- Pregnancy or breast-feeding.
- General medical condition contraindicating ECT.

### Study intervention

ECT will be performed with right unilateral electrode placement (RUL). The stimulation dose will be six times the seizure threshold based on titration during the first session. If patients do not show a clinical response after six treatments, electrode placement will be switched to bilateral electrode placement. Propofol or “ketofol” (esketamine + propofol) will be used as hypnotics. Time to stimulation will be 3–4 min after administration of the hypnotic. Succinylcholine will be used as a muscle relaxant in the absence of contraindicatons. Flumazenil will only be used if patients are treated with a lorazepam equivalent dose > 4 mg/day. Glycopyrronium bromide will be used to minimize hypersalivation. Esmolol and urapidil will be used to treat postictal hypertension. Electrode placement, stimulation dose, medications used during ECT, time to stimulation, postictal suppression index, midictal amplitude, maximum sustained coherence, maximum heart rate, motor seizure duration, and EEG seizure duration will be recorded in the eCRF.

During phase I, all patients will receive a full course of routine ECT (max. 18 sessions). In phase II, patients who are randomized to the experimental intervention group will additionally receive mECT. The mECT is administered according to a fixed schedule: Intervals will be one mECT per week for the first 6 mECTs, followed by one mECT every two weeks for 5 mECTs and then one mECT every three weeks for 4 mECTs (until week 28).

All patients in phase II will receive treatment according to current guidelines, including a stable continuation of the initial antipsychotic treatment (that was given concurrently to the acute ECT course in Phase I).

The stabilization period before the start of phase I will serve to implement any major changes to the medication that might be necessary before the initiation of the ECT series.

There are no restrictions regarding concomitant medication and therapy during study participation.

Adverse events (AE) will be asked for at each contact between the responsible investigator and the patient. AEs will be reported with patient ID, start and end date, description, grading, seriousness, relatedness, action taken and outcome.

### Statistical procedures

The primary analysis population will be the intention-to-treat (ITT) population consisting of all randomized patients, where each subject will be analyzed within the randomized group regardless of the actual treatment. Baseline is defined as last assessment before randomization. The primary analysis will be conducted when all patients completed visit 3 of the trial. An additional analysis will take place when all patients completed the follow up. SAS version 9.4 or higher will be used for analyses.

In the primary analysis, the time to relapse will be analyzed with a mixed cox regression model with planned treatment, strata (clozapine taken Yes/No) and BPRS at baseline as fixed effects and site as random effect in all patients randomized (ITT). Patients with missing information (including death) will be considered as relapses at the time point of last information. The occurrence of other intercurrent events is considered irrelevant in defining relapse (treatment policy strategy). Patients without relapse until visit 3 will be censored at visit 3 (due to possible change of intervention). The middle time point between date of awareness and the prior visit will be used to calculate the time to relapse.

The treatment effect will be tested using a two-sided alpha of 0.05 and in addition, a 95%-confidence interval will be calculated.

As secondary analyses, the number of relapse free patients at the end of phase II will be analyzed by a mixed logistic regression model (two-sided alpha = 0.05) with planned treatment, strata and BPRS at baseline as fixed effects and site as random effect in all patients randomized (ITT).

BPRS, GAF, PANSS, HAMD, NCRS-dv, Q-LES-Q-18, SSMIS-SF and stigma-stress-scale will be categorized considering relapses and dropouts as additional categories. These will be tabulated using standard descriptive measures including number of non-missing values, mean, standard deviation, extrema and quartiles.

Furthermore, to analyze BPRS, GAF, PANSS, HAMD, NCRS-dv, Q-LES-Q-18, SSMIS-SF and stigma-stress-scale per time-point linear mixed models with the respective scale at baseline, strata and treatment as fixed effects and site as random effect will be used.

An explorative analysis will be carried out for all recruited patients, where a logistic regression will be performed for the rate of inclusion in the randomized trial on baseline criteria. This way, patients likely to profit from a first session of ECT can be determined based on clinical criteria.

As a safety analysis, frequencies of patients experiencing at least one adverse event (AE) will be displayed. MMSE and THINC-it will be analyzed by group using standard descriptive measures.

As sensitivity analysis, the date of awareness will be used to calculate the time to relapse. Furthermore, the primary analysis is repeated using a log-rank-test.

Subgroup analyses by academic/non-academic site and strata will be carried out.

### Sample size / power calculation

Relapse rates after 6 months of treatment in phase II are estimated to be 90% in the TAU arm and 40% in the mECT + TAU arm based on a clinical trial with a similar study design, a systematic review and a recent open label CRS mECT study [24, 25, 41]. In this calculation, more conservative relapse rates of 85% (TAU) and 45% (mECT + TAU) are assumed; although most evidence indicates even lower relapse rates for mECT + TAU, but our patients might be more severely affected treatment resistant patients. We are aiming for sufficient power for the composite endpoint strategy where missing values are treated as relapses, which increases the relapse rates. With expected rates of missing values of 15% (TAU) and 30% (mECT + TAU) due to higher inconvenience with mECT + TAU, the relapse rates for planning the sample size are 87.25% (TAU) and 61.5% (mECT + TAU). This yields hazard rates of 0.3433 and 0.1591 and a hazard ratio of 0.4634, which in this case is a conservative approach.

Including 84 patients (42 per group) in phase II, a difference could be found with a power of 0.85 using a cox regression with alpha = 0.05 (two-sided) and considering the reduced effect due to missing values as specified above. Even if the expected hazard rate in the MECT + TAU arm is 0.17 ( ≙ 63.9%) e.g. due to lower effects or due to a bias because of different observation schedules, the power is still ~ 80%. (The necessary sample size was simulated using R.) 140 patients must be recruited in phase I, if 60% of patients are responders and willing to participate in phase II of the trial.

## Discussion

In this multi-center trial, we aim to examine the effectiveness of mECT in patients with CRS who improved after a course of routine ECT. If mECT will lead to a longer time to relapse and/or to a higher proportion of relapse-free patients compared to those undergoing treatment as usual, this trial would have an enormous impact on therapeutic strategies for “treatment-resistant” patients and would induce a profound change of current treatment guidelines, where ECT still ranks at the level of ultima ratio, despite accumulating evidence suggesting otherwise.

All procedures performed during this study are routine clinical procedures and are part of German guidelines. Of course, all side effects will be recorded. Severe side effects are not expected [42], since they are known to be extremely rare (and not study related).

The most common side effect in phase I will be cognitive side effects due to ECT. These will be monitored not only by MMSE, but also by THINC-it and by clinical observation. THINC-it has been validated multiple times in the past [36], especially for treatment-related changes of cognitive function in both depression [37] and SZ [38]. This monitoring will continue in phase II as well, although significant differences between groups are not expected to occur during this phase. Typically, cognitive side effects after an acute ECT series subside after about 15 days [43]. There is no evidence for further decline of cognitive function with maintenance ECT [44], but evidence of further improvement.

Participants might personally benefit from study participation due to closer and more standardized monitoring compared to routine clinical practice. Future patients with clozapine-resistant SZ will benefit from the study results, as they will most likely lead to a change in guidelines for treatment-resistant SZ.

## Electronic supplementary material

Below is the link to the electronic supplementary material.

Supplementary Material 1

Supplementary Material 2

## Data Availability

No datasets were generated or analysed during the current study.

## Abbreviations

AE: Adverse Event
BPRS: Brief Psychiatric Rating Scale
CRS: Clozapine-resistant schizophrenia
eCRF: Electronic Case Report Form
ECT: Electroconvulsive Therapy
GAF: Global Assessment of Functioning
HAMD: Hamilton Rating Scale for Depression
ITT: Intention-to-treat
mECT: Maintenance Electroconvulsive Therapy
MMSE: Mini Mental State Examination
NCRS: Northoff Catatonia Rating Scale
PANSS: Positive and Negative Symptom Scale
Q-LES-Q-18: Abbreviated Quality of Life Enjoyment and Satisfaction Questionnaire
RCT: Randomized controlled trial
REDCap: Research Electronic Data Capture
RUL: Right unilateral electrode placement
SSMIS-SF: Self-Stigma of Mental Illness Scale– Short Form
SZ: Schizophrenia
TAU: Treatment as usual
