# Supplementary material for: Study protocol of a German multi-center, observer-blind, randomized, and actively controlled parallel-group trial comparing maintenance electroconvulsive therapy to treatment as usual for relapse prevention in clozapine resistant schizophrenia
Source: BMC Psychiatry. 2025 May 26;25:536. doi: 10.1186/s12888-025-06990-2 (PMC12105297; doi:10.1186/s12888-025-06990-2)
Supplement: Supplementary file 1 — Supplementary Material 1 [file 12888_2025_6990_MOESM1_ESM.docx]

STUDY PROTOCOL

| Maintenance ElectroConvulsive Therapy in clozapine RESISTant schizophrenia  – the MECT-RESIST Trial – | |
| --- | --- |
| Coordinating Investigator Name and Address: | Prof. Dr. med. Alexander Sartorius  Central Institute of Mental Health  Department of Psychiatry and Psychotherapy, Medical Faculty Mannheim, University of Heidelberg  J5 - 68159 Mannheim  Tel.: 0621/1703-2984  Fax: 0621/1703-3165  E-mail: alexander.sartorius@zi-mannheim.de |
| Co-Coordinating Investigator Name and Address: | Prof. Dr. med. Robert Christian Wolf  Heidelberg University  Department of General Psychiatry  Voßstraße 4 \| 69115 Heidelberg  Tel.: 0621/ 56-4405  Fax: 0621/ 56-4481  E-mail: Christian.Wolf@med.uni-heidelberg.de |
| Study Registry Number (clinicaltrials.gov): | NCT06456983 |

| CONFIDENTIAL: This protocol contains confidential information and is intended solely for the guidance of the study. This protocol may not be disclosed to parties not associated with the study or used for any purpose without the prior written consent of the coordinating investigator. |
| --- |

# PROTOCOL SIGNATURE PAGE

The present study protocol was subject to critical review and has been approved in the current version by the persons undersigned. The information contained is consistent with:

- the current risk-benefit assessment of the study intervention(s),
- the moral, ethical, and scientific principles governing clinical research as set out in the latest relevant version of Declaration of Helsinki, the principles of the guidelines of ICH Good Clinical Practice and the applicable legal and regulatory requirements.

The investigators will be supplied with details of any significant change of the benefit-risk-assessment of the study.

It will be ensured that the first subject is enrolled only after all ethical and regulatory requirements are fulfilled. Written consent from all subjects is received after detailed oral and written information. It will be confirmed that all study subjects will be informed on the type of encoding of their personal data (pseudonymization) and who receives or has access to such data. Subjects who do not agree to this data encoding and transfer will not be enrolled into the study. In this context it will be assured that all investigational sites comply with the local regulatory requirements for data protection.

Via current versions of the study protocol it will be ensured that all principal investigators are informed about results regarding the benefits and risks of the study.

| Date: |  | Signature: |  |
| --- | --- | --- | --- |
|  |  | Name (Print Name): | Prof. Dr. med. Alexander Sartorius |
|  |  | Function: | Coordinating Investigator |
|  |  |  |  |
| Date: |  | Signature: |  |
|  |  | Name (Print Name): | Prof. Dr. med. Robert Christian Wolf |
|  |  | Function: | Co-Coordinating Investigator |
|  |  |  |  |
| Date: |  | Signature: |  |
|  |  | Name (Print Name): | Dr. Bernadette Wendel |
|  |  | Function: | Biometrician |

# INVESTIGATOR SIGNATURE PAGE

I have read the above-mentioned study protocol and confirm that it contains all information to conduct the study. I pledge to conduct the study according to the protocol, the principles of the guidelines of ICH Good Clinical Practice and the applicable legal and regulatory requirements.

I confirm that I have not entered into any financial agreement for this study, whereby the value of compensation paid to me could affect the outcome of this study.

I will enroll the first subject only after all ethical requirements are fulfilled. I will obtain written consent for study participation from all subjects or the legal representative, respectively, after detailed oral and written information. I declare that all subjects or the legal representative, respectively, will be informed on the type of encoding their personal data (pseudonymization) and who receives or has access to their data. Subjects who do not agree or whose legal representative, respectively, does not agree to this data encoding and transfer will not be enrolled into the study. In this context I confirm that my investigational site complies with all local regulatory requirements for data protection.

I pledge to document and notify such events as described in the protocol.

I confirm and will ensure that all staff will be adequately trained to guarantee compliance to the study protocol incl. subsequent modifications, the study procedures and study specific duties and tasks. I will maintain a list specifying the tasks delegated to each team member.

I will retain all study-related documents and source data as described. I will provide a Curriculum Vitae (CV) before study start. I agree that the CV may be submitted to the responsible EC.

| Date: | _________________ | Signature: | ________________________ |
| --- | --- | --- | --- |
|  |  | Name (Print Name): | ________________________ |
|  |  | Function: | Principal Investigator |
|  |  | Site (Address) | ________________________ |
|  |  |  | ________________________ |
|  |  |  | ________________________ |

# TABLE OF CONTENTS

[PROTOCOL SIGNATURE PAGE 2](#_Toc177721784)

[INVESTIGATOR SIGNATURE PAGE 3](#_Toc177721785)

[TABLE OF CONTENTS 4](#_Toc177721786)

[PROTOCOL SYNOPSIS 6](#_Toc177721787)

[SCHEDULE OF ASSESSMENT 8](#_Toc177721788)

[ABBREVIATIONS 11](#_Toc177721789)

[**1 Introduction and Rationale** 12](#_Toc177721790)

[1.1 Scientific Background 12](#_Toc177721791)

[1.2 Study Rationale / Justification 12](#_Toc177721792)

[1.3 Risk Benefit Assessment 13](#_Toc177721793)

[**2 Study Objectives and Endpoints** 14](#_Toc177721794)

[2.1 Primary Objective and Primary Endpoint 14](#_Toc177721796)

[2.2 Secondary Objectives and Endpoints 14](#_Toc177721797)

[**3 Study Design** 15](#_Toc177721798)

[3.1 Planned Interim Analysis 16](#_Toc177721800)

[3.2 Overall Duration of the Study 16](#_Toc177721801)

[3.3 Duration of Study Participation for each Subject 16](#_Toc177721802)

[3.4 Measures to Minimize Bias 16](#_Toc177721803)

[3.4.1 Randomization 16](#_Toc177721804)

[3.4.2 Rater/Observer-Blinding 16](#_Toc177721805)

[**4 Subject Selection** 17](#_Toc177721806)

[4.1 Number of Subjects and Sites 17](#_Toc177721808)

[4.2 Inclusion Criteria 17](#_Toc177721809)

[4.3 Exclusion Criteria 17](#_Toc177721810)

[**5 Study Intervention** 18](#_Toc177721811)

[5.1 Description of Study Intervention 18](#_Toc177721813)

[5.2 Concomitant Medication and Therapy 18](#_Toc177721814)

[**6 Study Visits and Investigations / Assessments** 19](#_Toc177721815)

[6.1 Planned treatment after End of Study Participation 20](#_Toc177721817)

[6.2 Assessments 20](#_Toc177721818)

[**7 Discontinuation and Early Termination** 22](#_Toc177721819)

[7.1 Temporary Discontinuation from Study Intervention 22](#_Toc177721821)

[7.2 Permanent Discontinuation from Study Intervention / Study 22](#_Toc177721822)

[7.2.1 Lost to Follow-Up 23](#_Toc177721823)

[7.3 Temporary Halt 23](#_Toc177721824)

[7.4 Early Termination of the Study 23](#_Toc177721825)

[7.5 Premature Closure of a Site 23](#_Toc177721826)

[**8 Adverse Events** 24](#_Toc177721827)

[8.1 Definitions 24](#_Toc177721829)

[8.1.1 Adverse Event 24](#_Toc177721830)

[8.1.2 Serious Adverse Event 25](#_Toc177721831)

[8.2 Characteristics of Adverse Events 25](#_Toc177721832)

[8.2.1 Grading of AEs 25](#_Toc177721833)

[8.2.2 Causal Relatedness 26](#_Toc177721834)

[8.2.3 Outcome of AEs 26](#_Toc177721835)

[8.2.4 Countermeasures 27](#_Toc177721836)

[8.3 Period of Observation and Documentation 27](#_Toc177721837)

[**9 Statistical Procedures** 28](#_Toc177721838)

[9.1 Definition of Study Population to be Analyzed 28](#_Toc177721840)

[9.2 Analysis Variables 28](#_Toc177721841)

[9.3 General Considerations 28](#_Toc177721842)

[9.4 Primary Analysis 28](#_Toc177721843)

[9.5 Secondary Analyses 28](#_Toc177721844)

[9.6 Interim Analyses 29](#_Toc177721845)

[9.7 Sensitivity Analyses 29](#_Toc177721846)

[9.8 Subgroup Analyses 29](#_Toc177721847)

[9.9 Sample Size / Power Calculation 29](#_Toc177721848)

[**10** **Data Management** 30](#_Toc177721849)

[10.1 Data Collection and Handling 30](#_Toc177721851)

[10.2 Data Coding 30](#_Toc177721852)

[10.3 Data Cleaning and Quality Checks 30](#_Toc177721853)

[**11 Archiving and Storage** 31](#_Toc177721854)

[**12 Regulatory, Ethical and Study Oversight Considerations** 31](#_Toc177721856)

[12.1 Compliance Statement 31](#_Toc177721858)

[12.2 Data Protection and Subject Privacy 31](#_Toc177721859)

[12.3 Approval of the Study 32](#_Toc177721860)

[12.4 Subject Information and Informed Consent 32](#_Toc177721861)

[12.5 Data Safety Monitoring Board (DSMB) 33](#_Toc177721862)

[**13 Quality Control and Quality Assurance** 33](#_Toc177721863)

[13.1 Quality Assurance 33](#_Toc177721865)

[13.2 Monitoring 33](#_Toc177721866)

[**14 Administrative Agreements** 34](#_Toc177721867)

[14.1 Financing of the Study 34](#_Toc177721869)

[14.2 Publication Policy 34](#_Toc177721870)

[**15 References** 35](#_Toc177721871)

# PROTOCOL SYNOPSIS

| **Full Title** | Maintenance ElectroConvulsive Therapy in clozapine RESISTant schizophrenia |
| --- | --- |
| **Study Code** | MECT-RESIST |
| **Rationale** | Schizophrenia is one of the most severe and costly mental disorders in terms of human suffering and societal expenditure. About 15-30% of patients do not respond to all known antipsychotics, including clozapine, the current gold-standard in these cases. Electroconvulsive therapy (ECT) is well known to be highly effective in clozapine-treatment-resistant schizophrenia (CRS), and synergistic effects of clozapine and ECT have been demonstrated. However, relapse rates after successful courses of ECT are still very high, and evidence for maintenance ECT (mECT) in CRS is scarce at best. In our multi-center trial, we aim to examine the effectiveness of mECT in treatment-resistant patients with schizophrenia who improved after a course of routine ECT.  The scientific aim of the study is to test the hypothesis that maintenance ECT (mECT) plus treatment as usual (TAU) (intervention group) is superior to TAU alone (control group) for relapse prevention in CRS. The effectiveness of mECT will be examined by comparing time to relapse in the intervention group with the control group expecting a longer time to relapse in the intervention group. Secondarily, a lower number of patients with relapse is expected in the intervention group compared to the control group. Further secondary objectives are to test the hypotheses that the global level of functioning and quality of life will increase, and that depression, overall symptoms of the schizophrenic syndrome, concomitant catatonic symptoms, stress and self-stigmatization will decrease compared to the control group. It is also hypothesized that cognitive performance will improve over the course of the mECT. |
| **Primary Objective and Endpoint** | To compare after a successful series of routine electroconvulsive therapy (ECT) the overall effectiveness (time to relapse) of maintenance ECT (mECT) plus treatment as usual (TAU) versus TAU only.  Endpoint: Time to relapse (relapse defined as Brief Psychiatric Rating Scale (BPRS) ≥20% higher than individual BPRS at start of phase II at any following study visit OR any unscheduled readmission due to a worsening of psychiatric symptoms OR any unscheduled visit with an BPRS ≥20% higher than individual BPRS at start of phase II). The BPRS is evaluated by an unblinded rater at site and a blinded central rater. Readmission is decided by unblinded non-study related staff. The first relapse decision is used for analysis. |
| **Main Secondary Objectives and Endpoints** | 1. To compare side effect profile and safety between mECT plus TAU and TAU only 2. To compare psychopathological changes: mECT plus TAU versus TAU only   Endpoints:  - Number of relapse free subjects at the end of phase II  - BPRS at the end of phase II  - GAF, PANSS, HAMD, NCRS-dv, Q-LES-Q-18, SSMIS-SF, stigma-stress-scale at the end of phase II  - BPRS, GAF, PANSS, HAMD, NCRS-dv, Q-LES-Q-18,  SSMIS-SF, stigma-stress-scale at the end of follow-up  Assessment of safety:  Cognitive side effects profile (MMSE, THINC-integrated tool [THINC-it])  and safety. |
| **Study Design** | This is a multi-center, observer-blind, randomized, and actively controlled parallel-group clinical trial. |
| **Sample Size** | To be assessed for eligibility (n = 300)  To be allocated to trial (n = 140 in phase I)  To be analyzed (n = 84 randomized in phase II) |
| **Study Population** | Subjects (aged 18-65 years) with current diagnosis of schizophrenia according to Diagnostic and Statistical Manual of Mental Disorders, Fifth Edition (DSM-5), BPRS total score > 45 and history of clozapine resistant schizophrenia (CRS), which will include treatment-resistant schizophrenia with clozapine intolerance or absolute contraindications for clozapine. |
| **Interventions** | Experimental intervention:  All included subjects will be on a stable antipsychotic drug regime for 2 weeks before inclusion. All subjects will enter phase I and will receive a full course of routine ECT while being on stable antipsychotic medication. All ECT-responders (subjects with improvement of 30% or more on BPRS will enter phase II and will be randomly assigned to the experimental intervention (mECT plus TAU) or the control intervention (TAU) which both last 28 weeks. Non-responders (subjects without improvement of at least 30 % on BPRS scale) will not enter phase II.  Control intervention:  Subjects randomized to TAU only will continue on a stable drug regime for the next 28 weeks, but will not receive mECT. The number of clinical and study visits will be identical in both arms.  Intervention per patient:  Max. 35 weeks in total including a 1-week stabilization period, max. 6  weeks of routine ECT (maximal 18 sessions), and an active intervention  period of 28 weeks (15 ECT sessions).  Follow-up period: 12 months with or without maintenance ECT as determined by patients  Experimental and / or control off label or on label in Germany:  Both are on label in Germany |
| **Number of Sites** | 14 |

# SCHEDULE OF ASSESSMENT

| **Phase (duration)** | **0** | **I** | | **II** | | | | | | **Follow-Up** | | **Unscheduled**  **Visit** |
| --- | --- | --- | --- | --- | --- | --- | --- | --- | --- | --- | --- | --- |
|  | max. 1 week | Initial ECT series  (max. 6 weeks and max. 18 ECTs are allowed) | | 28 weeks | | | | | | 12 months | |  |
| **Visit** | **Screening/ inclusion** | **1** | **2** | **2a** | **2b** | **2c** | **2d** | **2e** | **3** | **4a** | **4b** |  |
| **Time** | Before start of stabilization*  (max. 7 days before visit 1) | 1-2 day(s) before the first ECT | 1 day after last ECT (week 0) | 3rd calendar week ** or  on the day of the 3rd mECT | 6^th^ calendar week after V2 or  on the day of the 6th mECT | 10^th^ calendar week after V2 or on the day of the 8th mECT | 14^th^ calendar week after V2 or on the day of the 10th mECT | 19^th^ calendar week after V2 or on the day of the 12th mECT | 28^th^ calendar week after V2  or on the day of the last mECT | 6 mo. after visit 3 | 12 mo. after visit 3 |  |
| Informed Consent | **x** |  |  |  |  |  |  |  |  |  |  |  |
| In-/Exclusion Criteria | **x** |  |  |  |  |  |  |  |  |  |  |  |
| Sociodemographics^1^ |  | **x** |  |  |  |  |  |  |  |  |  |  |
| Medical History |  | **x** |  |  |  |  |  |  |  |  |  |  |
| Concomitant Medication / Therapy |  | **x** | **x** | **x** | **x** | **x** | **x** | **x** | **x** | **x** | **x** | **x** |
| Vital Signs^2^ |  | **x** |  |  |  |  |  |  | **x** |  | **x** | **x** |
| Randomization^3^ |  |  | **x** |  |  |  |  |  |  |  |  |  |
| Adverse events |  |  |  | **x** | **x** | **x** | **x** | **x** | **x** | **x** | **x** | **x** |
| **Intervention** |  |  |  |  |  |  |  |  |  |  |  |  |
| TAU or mECT + TAU |  |  |  | **x**^4^ | **x**^4^ | **x**^4^ | **x**^4^ | **x**^4^ | **x**^4^ | **x**^5^ | **x**^5^ |  |
| **Questionnaires/tests** |  |  |  |  |  |  |  |  |  |  |  |  |
| Brief Psychiatric Rating Scale |  | **x** | **x** | **x** | **x** | **x** | **x** | **x** | **X** | **x** | **x** | **x** |
| Global Assessment of Functioning |  | **x** | **x** | **x** | **x** | **x** | **x** | **x** | **x** | **x** | **x** | **x** |
| Positive and Negative Symptom Scale |  | **x** | **x** | **x** | **x** | **x** | **x** | **x** | **x** | **x** | **x** | **x** |
| Hamilton Rating Scale for Depression |  | **x** | **x** | **x** | **x** | **x** | **x** | **x** | **x** | **x** | **x** | **x** |
| Mini Mental State^6^ Examination |  | **x** | **x** | **x** | **x** | **x** | **x** | **x** | **x** | **x** | **x** | **x** |
| THINC-it ®^6^ |  | **x** | **x** | **x** | **x** | **x** | **x** | **x** | **x** | **x** | **x** | **x** |
| Northoff Catatonia Rating Scale (German version) |  | **x** | **x** |  |  |  |  |  | **x** | **x** | **x** | **x** |
| Q-LES-Q-18 |  | **x** | **x** |  |  |  |  |  | **x** | **x** | **x** | **x** |
| Self-Stigma of Mental Illness Scale – Short Form |  | **x** | **x** |  |  |  |  |  | **x** | **x** | **x** | **x** |
| Stigma-Stress-Scale |  | **x** | **x** |  |  |  |  |  | **x** | **x** | **x** | **x** |

ECT: Electroconvulsive Therapy; mECT: Maintenance Electroconvulsive Therapy; TAU: treatment as usual; THINC-it: THINC integrative tool; Q-LES-Q-18: Abbreviated Quality of Life Enjoyment and Satisfaction Questionnaire

*Stabilization: implementation of any major medication changes before the initiation of the ECT series

** The times indicated as weeks refer to the TAU group, however the respective mECT should be scheduled within the respective week. Visit 2 is regarded to take place in week 0, e.g. calendar week 20. For the patients receiving mECT, the first mECT should be scheduled in the next calendar week, e.g. calendar week 21, and the 3^rd^ mECT (Visit 2a) should take place in calendar week 3 after V2, e.g. calendar week 23. A calendar week is from Monday to Sunday. Two mECTs cannot be performed in the same calendar week

^1^ Sociodemographics: Age, sex, education, marital status, residence

^2^ Vital signs: height, weight

^3^ All baseline characteristics (including vital signs, medical history, prior medication, …) must be assessed **before** randomization. Randomization should be carried out only in subjects with improvement of 30% or more on the Brief Psychiatric Rating Scale (BPRS).

^4^ According to randomization

^5^ Treatment depending on patients’ choice

^6^ Mini-Mental State Examination (MMSE) and Thinc-it must be performed before mECT is administered.

# ABBREVIATIONS

AE Adverse Event

BPRS Brief Psychiatric Rating Scale

CRF Case Report Form

CTCAE Common Terminology Criteria for Adverse Events

CTFG Clinical Trial Facilitation Group

CV Curriculum Vitae

DBL Data Base Lock

DMP Data Management Plan

DSMB Data Safety Monitoring Board

DVP Data Validation Plan

eCRF electronic Case Report Form

ECT Electroconvulsive Therapy

EU European Union

FAS Full Analysis Set

FSI First Subject In

GAF Global Assessment of Functioning

GCP Good Clinical Practice

GDPR General Data Protection Regulation

HAMD Hamilton Rating Scale for Depression

IC Informed Consent

ICH International Council on Harmonization of Technical Requirements for Registration of Pharmaceuticals for Human Use

ICH GCP ICH harmonized tripartite guideline on GCP

IEC Independent Ethics Committee

IIT Investigator-Initiated Trial

ISF Investigator Site File

ISRCTN International Standard Randomized Controlled Trial Number

KKS Coordination Centre for Clinical Trials (Koordinierungszentrum für Klinische Studien)

LDR Legally Designated Representative

LSLV Last Subject Last Visit

mECT Maintenance Electroconvulsive Therapy

MMSE Mini Mental State Examination

NCRS Northoff Catatonia Rating Scale

PANSS Positive and Negative Symptom Scale

PD Protocol Deviation

PI Principal Investigator

Q Quarter (time span)

Q-LES-Q-18 Abbreviated Quality of Life Enjoyment and Satisfaction Questionnaire

REDCap Research Electronic Data Capture

RDE Remote Data Entry

SC Steering Committee

SOP Standard Operating Procedure

SSMIS-SF Self-Stigma of Mental Illness Scale – Short Form

TAU Treatment as usual

TMF Trial Master File

# Introduction and Rationale

## Scientific Background

Schizophrenia is one of the most severe and costly mental disorders in terms of human suffering and societal expenditure (1-5). Life-time prevalence of schizophrenia is about 0.5–1.0%. Sustained recovery occurs in less than 14% within the first five years following a psychotic episode. Longer-term outcomes may be marginally better: a large international 25-year follow-up study reported an additional 16% with late-phase recovery. Throughout Europe, less than 20% of people with schizophrenia are employed (6). Years of potential life lost typically range between 10-20 years (7).

One major problem is that a significant proportion of patients - about 15-30 % - do not respond to all known antipsychotics, including clozapine, which is currently considered the gold-standard for treatment-resistant schizophrenia (8). These patients are among the most debilitated patients with a poor outcome prognosis in terms of medical and social rehabilitation. For these severely ill patients with persistent positive symptoms there are no further evidence-based alternatives or augmentation strategies. Hence, recent Cochrane database reviews stated (9, 10) that the quality of extant studies is too poor to recommend any intervention additional to clozapine and that new, properly conducted, randomized controlled trials independent from the pharmaceutical industry need to be performed to help these most severely impaired patients (9, 10).

Although ECT was initially used to treat schizophrenia and is recommended in national treatment

guidelines and by our national psychiatric associations (11, 12), it is nowadays by far underused in the therapy of schizophrenia in many countries, including Germany. This may be due to its stigma, but it is also a consequence of the development of antipsychotic medication, which however possesses its own inherent limitations and disadvantages. ECT is well known to be effective in treatment resistant schizophrenia and CRS (see e.g. (13-18)), and synergistic effects of clozapine and ECT have been demonstrated (19-23). However, relapse rates after successful courses of ECT are very high (16), and evidence for the use of maintenance ECT (mECT) in treatment resistant schizophrenia (24) or CRS (25) is scarce at best. At present, there are only case reports and a limited number of retrospective studies demonstrating that the clinical severity of the illness and re-hospitalizations can be significantly reduced with mECT (15, 25, 26).

## Study Rationale / Justification

In this multi-center trial, we aim to examine the effectiveness of mECT in treatment-resistant subjects with schizophrenia who improved after a course of routine ECT. If mECT will lead to a longer time to relapse and/or to a higher proportion of relapse-free subjects compared to those undergoing treatment as usual, this trial would have an enormous impact on therapeutic strategies for "treatment-resistant" subjects and would induce a profound change of current treatment guidelines, where ECT still ranks at the level of ultima ratio, despite accumulating evidence suggesting otherwise.

## Risk Benefit Assessment

All procedures performed during this study are routine clinical procedures and are mentioned within German guidelines. Of course, all side effects will be recorded. Severe side effects are not expected (27), since they are known to be extremely rare (and not study related).

The most common side effect in phase one will be cognitive side effects due to ECT. These will be monitored not only by MMSE, but also by THINC-it and by clinical observation. THINC-it has been validated multiple times in the past (28), especially for treatment-related changes of cognitive function in both depression (29) and schizophrenia (30). Of course, this monitoring will continue in phase two as well, although significant differences between groups are not expected to occur during this phase. Typically, cognitive side effects after an acute ECT series subside after about 15 days (31). There is no evidence for further decline of cognitive function with maintenance ECT (32), but evidence of further improvement.

Participants might personally benefit from study participation due to closer and more standardized monitoring compared to routine clinical practice. Future patients with clozapine-resistant schizophrenia will benefit from the study results, as they will most likely lead to a change in guidelines for treatment-resistant schizophrenia.

# Study Objectives and Endpoints


## Primary Objective and Primary Endpoint

The main purpose of the study is to show superiority of maintenance electroconvulsive therapy (mECT) plus treatment as usual (TAU) versus TAU only.

The primary endpoint is the time to relapse for which relapse is defined as Brief Psychiatric Rating Scale (BPRS) ≥20% higher than individual BPRS at start of phase II at any following study visit or any unscheduled readmission due to a worsening of psychiatric symptoms or any unscheduled visit with an BPRS ≥20% higher than individual BPRS at start of phase II.

The BPRS is evaluated by an unblinded rater at the site and a blinded central rater. Hospital readmission is decided by unblinded non-study related staff. The first relapse decision is used for analysis.

If the condition of a patient is worsening (assessed by unblinded staff), the BPRS assessment is videotaped for the evaluation by the blinded rater.

The time to relapse is the most important outcome parameter, when the effectiveness of interventions in subjects with schizophrenia is investigated. The BPRS is a widely used instrument in psychiatry for the assessment of psychotic symptoms and has been implemented in previous clinical trials and large-scale investigations (33). The instrument has been internationally validated and proven to be sensitive to change.

## Secondary Objectives and Endpoints

The secondary objectives are to compare side effect profile and safety as well as psychopathological changes between mECT plus TAU versus TAU only.

The secondary endpoint for treatment efficacy is the proportion of relapse free subjects at the end of phase II.

Further secondary endpoints are:

- BPRS
- Global assessment of functioning (GAF) allowing a general (34) and PANSS allowing a differentiated view on specific symptom dimensions (35)
- HAMD (36) monitoring depression, since ECT and mECT have a well-known positive influence on mood
- The Northoff catatonia rating scale (NCRS-dv) (37) assessing the full spectrum of catatonic symptoms
- Q-LES-Q-18, an abbreviated and validated Quality of Life Enjoyment and Satisfaction Questionnaire for subjects with schizophrenia (38)
- The Self-Stigma of Mental Illness Scale – Short Form (SSMIS-SF) (39) and the Stigma-Stress-Skala (40) are used for self-labeling and stigma
- MMSE (41) and THINC-it (28-30) monitoring cognition

All secondary endpoints listed above are analyzed at the end of phase II and at the end of follow-up.

Safety will be also determined by the frequency of adverse events from randomization up to the last follow-up visit.

# Study Design

This is a German multi-center, observer-blind, randomized, and actively controlled parallel-group study to compare after a successful series of routine electroconvulsive therapy (ECT) the overall effectiveness of maintenance ECT (mECT) plus treatment as usual (TAU) versus TAU only in subjects aged from 18 to 65 years with the indication of clozapine resistant schizophrenia.

A schematic of the study design is shown below:


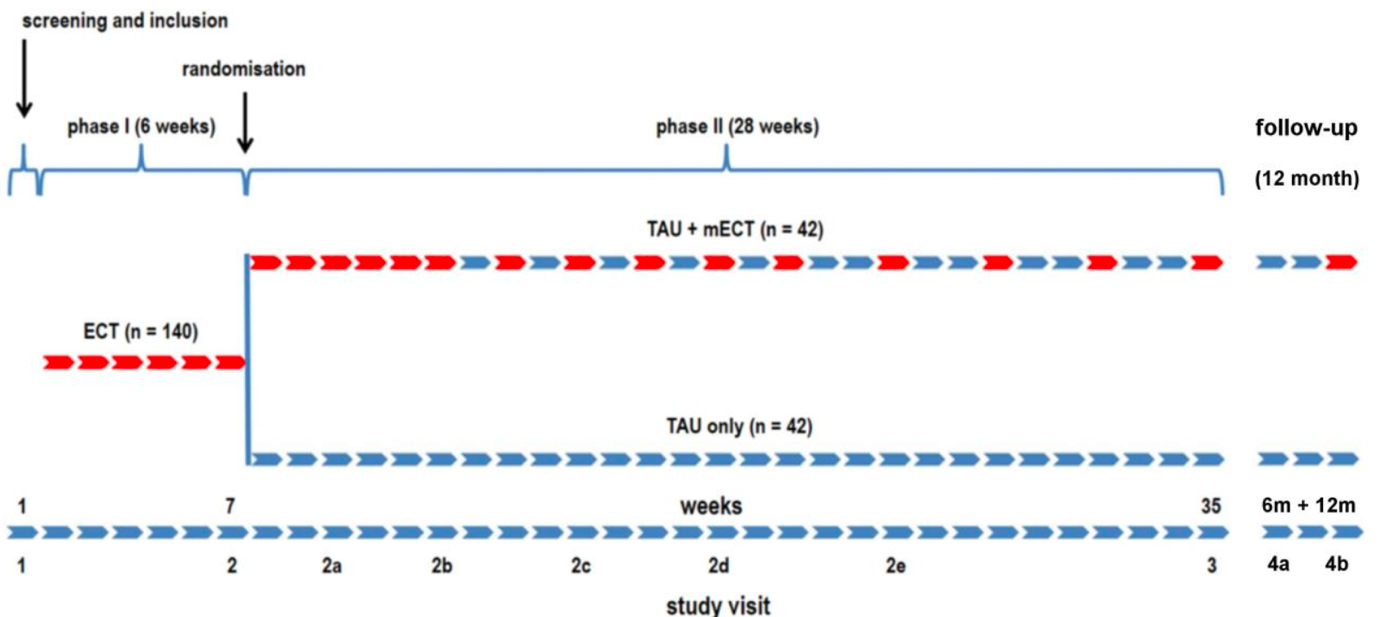


**Phase I:** Subjects will be included in the study according to the inclusion and exclusion criteria and will receive an ECT series with a maximum of 18 ECT treatments based on a clinical indication for ECT or until there is a clinically sufficient response to treatment.

**Phase II:** Subjects who respond to ECT in phase I (BPRS score < 70% of BPRS at visit 1) meet the inclusion criteria for phase II of the study. After completing the ECT series in phase I and before starting phase II, subjects are randomized to receive either maintenance ECT plus standard treatment or standard treatment alone.


## Planned Interim Analysis

No interim analysis is planned.

## Overall Duration of the Study

The study will be conducted over a period of 54 months.

The duration of the study may be extended and additional sites may be added depending on the observed rate of recruitment.

| Total study duration: | 54 months |
| --- | --- |
| Duration of the clinical phase: | 39 months |
| FSI (First Subject In): | Q1/2025 |
| LSLV (Last Subject Last Visit): | Q2/2028 |
| Statistical analyses completed: | Q4/2028 |

The study end is defined as “last subject last visit” (LSLV).

In case of an early termination of the study (see chapter 7.4), the date of the early termination will be the date for end of study.

## Duration of Study Participation for each Subject

The study consists of:

- Screening and stabilization phase: max. 1 week
- Phase I (ECT): max. 6 weeks
- Phase II (maintenance ECT + TAU or TAU): 28 weeks
- Follow-up period: 12 months

In total the duration of the study for each subject is expected to be at maximum 20 months.

## Measures to Minimize Bias

- - 1. Randomization

Subjects will be randomized 1:1 by the site staff using REDCap (eCRF), which maintains allocation concealment by accepting subjects’ inclusion before assigning the treatment. Block randomization will be applied stratified by subjects taking clozapine at visit 2 and subjects not taking clozapine at visit 2 but not site in order to prevent predictability of allocation.

- - 1. Rater/Observer-Blinding

The interviews regarding BPRS will be videotaped at baseline (visit 2) as well as at any planned and/or unplanned visit at which a subject is experiencing a worsening of its condition. The videos will be edited to remove any hints about treatment, and brief (2–5 seconds), nonessential portions from all tapes will be erased to create 3–10 skips in a random fashion, thus balancing the presence of awkward skips that might be otherwise present mostly in the ECT tapes. An independent, blinded rater (psychologists and psychiatrists trained in the assessment of psychopathological symptomatology) will score the edited tapes.

The primary endpoint is combined endpoint of judgements by blinded and unblinded non-study related raters (see section 2.1). Strictly speaking, the study is only partially observer blind. Blinding of the observer at the site is impossible because the patients usually reveal their actual treatment during assessments. Therefore, it is justifiable from a medical point of view to call the study observer-blind.

# Subject Selection


## Number of Subjects and Sites

A total of 140 subjects will be enrolled in the study (phase I) and 84 subjects will be randomized (phase II), i.e. 42 subjects per treatment group (see chapter 9.9).

The study will be national and multicenter.

It is intended that the study will take place at approximately 14 German sites.

## Inclusion Criteria

Subjects meeting all of the following criteria will be considered for enrollment in the study:

- Current schizophrenia according to Diagnostic and Statistical Manual of Mental Disorders, Fifth Edition (DSM-5)
- BPRS total score > 45
- History of clozapine resistant schizophrenia (CRS), which will include treatment-resistant schizophrenia with clozapine intolerance or absolute contraindications for clozapine
- Age between 18 and 65 years

## Exclusion Criteria

Subjects presenting with any of the following criteria will not be included in the study:

- Diagnosis of DSM-5 major neurocognitive disorder (“dementia”), current severe substance-use disorder, affective disorders with psychotic symptoms or any personality disorder
- Inability to read/write German or inability to provide written informed consent
- Pregnancy or breast-feeding
- General medical condition contraindicating ECT

# Study Intervention


## Description of Study Intervention

**Electroconvulsive Therapy (ECT)**

ECT will be performed with right unilateral electrode placement (RUL). The stimulation dose will be six times the seizure threshold based on titration during the first session. If subjects do not show a clinical response after six treatments, electrode placement will be switched to bilateral electrode placement. Propofol or ketofol (esketamine + propofol) will be used as hypnotics. Time to stimulation will be 3-4 minutes after administration of the hypnotic. Succinylcholine will be used as a muscle relaxant in the absence of contraindicatons. Flumazenil will only be used if subjects are treated with a lorazepam equivalent dose > 4 mg/day. Glycopyrronium bromide will be used to minimize hypersalivation. Esmolol and urapidil will be used to treat postictal hypertension. Electrode placement, stimulation dose, medications used during ECT, time to stimulation, postictal suppression index, midictal amplitude, maximum sustained coherence, motor seizure duration, and EEG seizure duration will be recorded in the eCRF.

**Phase I:** All subjects receive a full course of routine ECT (max. 18 sessions; 3x/week)

**Phase II:** Subjects who are randomized to the experimental intervention group will additionally receive mECT. The maintenance ECT is administered according to a fixed schedule: Intervals will be one mECT per week for the first 6 mECTs, followed by one mECT every two weeks for 5 mECTs and then one mECT every three weeks for 4 mECTs (until week 28).

**Treatment-as-usual** (**TAU)**

All subjects in phase II will receive treatment according to current guidelines, including a stable continuation of the initial antipsychotic treatment (that was given concurrently to the acute ECT course in Phase I).

**Stabilization (before start of phase I)**

The stabilization period before the start of phase I will serve to implement any major changes to the medication that might be necessary before the initiation of the ECT series.

## Concomitant Medication and Therapy

There are no restrictions regarding concomitant medication and therapy during study participation.

# Study Visits and Investigations / Assessments

This section describes the procedures and assessments required to be performed at specified study visits as outlined in the schedule of assessments. For details on the individual assessments please refer to section 6.2.

The interventions/assessments will be recorded in the source documents and the eCRF.

**Screening Visit / stabilization phase**

- Review of inclusion/exclusion criteria
- Obtaining written informed consent

**Phase I**

- Study visits 1 and 2 with psychometric assessments
- Full course of routine ECT (max. 18 sessions; 3x/week)
- Randomization (at the end of visit 2) of subjects with improvement of 30% or more on the Brief Psychiatric Rating Scale (BPRS)

**Phase II**

- First mECT should be administered within 1 week after randomization
- Study visits 2a-e and 3 with psychometric assessments are done on the day of the third, sixth, eighth, tenth twelfth or fifteenth maintenance ECT, respectively, for the intervention group. Thinc-it and MMSE must be assessed before the treatment is administered, the remaining psychometric assessments can be performed before or after mECT is administered.
- Intervention group: TAU + mECT
- Control group: Only TAU

**Visit 3**

Study participation can regularly end at study visit 3.

If the study participation ends prematurely (before visit 3), see section 7.3, the assessments according to visit 3 should be done.

**Follow-Up period**

- Study visits 4a and b with psychometric assessments
- Treatment with or without mECT depending on patient’s choice regardless of randomization


## Planned treatment after End of Study Participation

Further treatments after the subject’s end of study visit should be performed according to local standard procedures at each site, chosen at the discretion of the treating psychiatrist.

## Assessments

- **Sociodemographic:** Age, sex, marital status, education (in years including: school degree (Hochschulreife/Abitur, Realschulabschluss, Hauptschulabschluss), university degree (bachelor), higher education (master, PhD)) and residence (city with 100.000 inhabitants, town with 10.000 inhabitants and rural area)
- **Medical history:**

This includes psychiatric history including number of psychotic episodes, prior treatments and concomitant psychiatric disorders, other clinically significant diseases, surgeries, previous medical procedures, smoking history, use of alcohol and other drugs, and all current medications (e.g., prescription drugs, over-the-counter drugs, herbal or homeopathic remedies, and nutritional supplements).

- **Vital signs/physical examination:** Height, weight
- **Concomitant medication:** Medication taken during subject’s enrollment will be documented.
- **AEs:**

AEs will be asked for at each contact between the responsible investigator and the subject. AEs will be reported with subject ID, start and end date, description, grading, seriousness, relatedness, action taken and outcome.

- Treatment (mECT) parameters (see section 5.1)

The following **questionnaires** will be completed by the investigator during the study visits:

- **Brief Psychiatric Rating scale (BPRS):** A 18-item rating scale to measure psychiatric symptoms such as depression, anxiety, hallucinations and unusual behavior using a seven-item Likert scale. The BPRS will be assessed by means of a clinical interview that will be recorded on video and rated by a blinded rater and unblinded raters at the sites.
- **Global Assessment of Functioning (GAF):** A numeric scale to rate the social, occupational, and psychological functioning of an individual. Scores range from 100 (extremely high functioning) to 1 (severely impaired).
- **Positive and Negative Syndrome Scale (PANSS):** A numeric scale used for measuring symptom severity of patients with schizophrenia. The subject is rated from 1 to 7 on 30 different symptoms. The total score ranges from 30 to 210.
- **Mini Mental State Examination (MMSE):** The examination will be performed to screen for cognitive impairment and associated reduction. It includes tests of orientation, attention, memory, language and visual-spatial skills. The patient can reach a maximum of 30 points (no cognitive impairments) in MMSE total score.
- **17-item Hamilton Rating Scale for Depression (HAMD):** A 17-item questionnaire used to provide an indication of depression. Items are scored on a 5-point Likert-type scale. A score of 0–7 is considered to be normal, while a score of >23 is considered severe.
- **Northoff Catatonia Rating Scale (German version) (NCRS-dv):** The NRCS-dv will be used to assess the full spectrum of catatonic symptoms. It consists of 40 items describing motor (13 items), affective (12 items) and behavioral (15 items) catatonic symptoms.

The following **self-assessment scales** will be completed by subjects digitally during the study visits under the supervision of a study team member:

- **Quality of Life Enjoyment and Satisfaction Questionnaire (Q-LES-Q-18):** The Q- LES-Q-18 is an abbreviated and validated Quality of Life Enjoyment and Satisfaction Questionnaire for patients with schizophrenia.
- **THINC-it ®:** Online-Tool for Cognitive Assessment and Measurement which includes 4 objective cognitive tests (adapted from choice reaction time, 1-back working memory task, symbol digit coding, and Trails-B) and a subjective cognitive questionnaire. Results are displayed as standard deviation from the results of healthy age-, sex- and education-matched comparison individuals. <https://progress.im/en/content/download-thinc-it%C2%AE-tool>
- **Self-Stigma of Mental Illness Scale – Short Form (SSMIS-SF):** A short form of the 40-item Self-Stigma of Mental Illness Scale consisting of 20 items.
- **Stigma-Stress-Skala:** An 8-item-scale consisting of 4 items assessing perceived harmfulness of mental health stigma and 4 items assessing perceived resources to cope with stigma, with items scored from 1 to 7. A single stress appraisal is computed by subtracting perceived resources from perceived harmfulness. A higher difference score indicates the appraisal of stigma as stressful and as exceeding personal coping resources.

# Discontinuation and Early Termination

Any subject can withdraw from the study intervention or the study verbally or in writing at any time without personal disadvantages and without having to give a reason. However, the investigator should make a reasonable effort to ascertain possible reasons, while fully respecting the subject's rights. Specifically, the subject must not be coerced or unduly influenced to continue participation. Any given reason should be specified in the subjects file and in the CRF.

The investigator can also discontinue the study intervention after considering the risk-to-benefit ratio, if he / she no longer considers the treatment justifiable. The date of and the primary reason for withdrawal (one primary reason must be determined), as well as the observations available at the time of withdrawal are to be specified in the CRF.


## Temporary Discontinuation from Study Intervention

The study intervention is allowed to be postponed once for at most one calendar week but not to skip one.

Visit 2 is regarded to take place in week 0, e.g. calendar week 20. For the patients receiving mECT, the first mECT should be scheduled in the next calendar week, e.g. calendar week 21, and the 3rd mECT (Visit 2a) should take place in calendar week 3 after V2, e.g. calendar week 23 and so forth. A calendar week is from Monday to Sunday.

It is allowed to postpone one mECT for at most one calendar week, e.g. if the mECT is planned in the calendar week 5, it is allowed to perform it in week 6 but neither in week 7 nor week 4. Thereafter, all mECTs have to be shifted, e.g. if the mECT is performed in week 6 instead of week 5, the next mECT has to take place in week 7. Therefore, the last mECT takes place in calendar week 29 after V2.

It is not allowed to postpone mECTs twice, so that the last mECT would take place 30 weeks after V2.

It is not allowed to receive two mECTs within one calendar week.

## Permanent Discontinuation from Study Intervention / Study

Permanent discontinuation from study intervention is any discontinuation associated with the investigator’s or the subject’s definitive decision not to re-initiate the study intervention. The following criteria will lead to a permanent discontinuation from study intervention:

- Subject’s request, i.e. withdrawal of consent,
- Loss to follow up,
- Relapse (BPRS > 120% of baseline [visit 2]) during a study visit or during an unplanned visit
- Unplanned hospitalization due to worsening psychiatric symptoms.
- The treatment is postponed more than once for at most one week or the treatment is postponed more than one week.

All subjects who discontinue the study prematurely, a withdrawal examination at least with respect to the secondary endpoint is offered at any time up to week 28 of the subject study inclusion (time of randomization, Visit 2). The subject must be asked to consent to this last examination. The (oral) consent must be documented in the patient file. The withdrawal examination must be documented in the CRF.

For details concerning sample and statistical considerations see chapter 9.

- - 1. Lost to Follow-Up

Subjects will be considered lost to follow-up if they fail to show up for study visits and cannot be contacted by the site. Site personnel is expected to make diligent attempts to contact subjects who did not show up for a scheduled visit or were otherwise not available. These contact attempts should be documented in the subject’s medical record.

## Temporary Halt

A temporary halt of a study is defined as an unforeseen interruption not provided in the protocol but with the intention to resume it. If the reason for the temporary halt may have a negative effect on benefit-risk assessment, re-start is possible only after approval of a substantial modification.

## Early Termination of the Study

Early termination is defined as the premature end of a clinical study before at least the last patient has completed visit 3 .

The following reasons or events may result in an early termination:

- New findings on the study intervention lead to doubt as to the benefit-risk ratio;
- Subject enrollment is insufficient;
- DSMB recommends termination of the entire study or single treatment arms.

An early end of the study due to early inclusion of the total number of subjects is not considered an early termination. In the event of premature discontinuation of the study for any reason whatsoever, the regulatory authorities should be informed according to applicable regulatory requirements. In case of an early termination of the study, the date of early termination will be the date of end of study.

## Premature Closure of a Site

Premature closure of a single site by coordinating investigator may be considered for the following reasons:

• Study centers that fail to recruit any subject in the first 6 month after initiation.

• Study centers that fail to meet the minimum recruitment goal of 3 subjects within 12 months.

• Non-compliance with ICH-GCP, any provision of the study protocol or breach of the applicable laws and regulations of the investigator, sub-investigator or delegated staff.

The investigator may terminate participation in the study himself/herself if at his/her own discretion the site or the investigator becomes unable to perform or complete the study according to the agreement in place.

# Adverse Events


## Definitions

- - 1. Adverse Event

Following GCP, the definition of an adverse event (AE) is adapted as follows: Any untoward medical occurrence in a subject participating in a study and which does not necessarily have a causal relatedness with the study intervention. An AE can therefore be any unfavorable and unintended sign, symptom, or disease temporally associated with the study intervention, whether or not related to the study intervention.

An AE may be:

- New symptoms / medical conditions,
- New diagnosis,
- Intercurrent diseases and accidents,
- Worsening of medical conditions/ diseases existing before study start,
- Recurrence of disease,
- Increase of frequency or intensity of episodic diseases.

A pre-existing disease or symptom will not be considered an adverse event unless there will be an untoward change in its intensity, frequency or quality. This change will be documented by an investigator.

Surgical procedures themselves are not AEs; they are therapeutic measures for conditions that require surgery. The condition for which the surgery is required may be an AE. Planned surgical measures permitted by the study protocol and the condition(s) leading to these measures are not AEs, if the condition leading to the measure was present prior to inclusion into the study. In the latter case the condition should be reported as medical history.

AEs are classified as "non-serious" or "serious".

- - 1. Serious Adverse Event

Serious adverse event (SAE) is untoward medical occurrence that:

- Results in death,
- Is life-threatening (the term life-threatening refers to an event in which the subject was at risk of death at the time of event and not to an event which hypothetically might have caused death if it was more severe),
- Requires hospitalization or prolongation of existing hospitalization,
- Results in persistent or significant disability / incapacity*,
- Is a congenital anomaly / birth defect,
- Is otherwise medically relevant.

* Persistent or significant disability or incapacity means that there is a substantial disruption of a person's ability to carry out normal life functions. The irreversible injury of an organ function (e.g. paresis, diabetes, cardiac arrhythmia) fulfils this criterion.

Medical and scientific judgement should be exercised in deciding whether expedited reporting to coordinating investigator is appropriate in other situations - such as important medical events that may not be immediately life threatening or result in death or hospitalization but may jeopardize the subject or may require intervention to prevent one of the other outcomes listed above. These should also usually be considered serious (examples of such events are intensive treatment in an emergency room or at home for allergic bronchospasm; blood dyscrasias or convulsions that do not result in hospitalization).

However, since the following events do not change the ongoing benefit-risk assessment of the present study, they do not need to be reported as SAEs:

- Hospitalization aiming exclusively at diagnostic measures or due to technical, practical or social reasons, i.e. hospitalization without underlying adverse event

## Characteristics of Adverse Events

- - 1. Grading of AEs

The grading of AEs in this study will be carried out on the basis of the 5-grade scale defined in the CTCAE V 5.0:

| Grade 1: | Mild; asymptomatic or mild symptoms; clinical or diagnostic observations only; intervention not indicated. |
| --- | --- |
| Grade 2: | Moderate; minimal, local or non-invasive intervention indicated; limiting age-appropriate instrumental Activities of Daily Living (ADL)*. |
| Grade 3: | Severe or medically significant but not immediately life-threatening; hospitalization or prolongation of hospitalization indicated; disabling; limiting self-care ADL**. |
| Grade 4: | Life threatening consequences; urgent intervention indicated. |
| Grade 5: | Death related to AE. |

*Instrumental ADL refer to preparing meals, shopping for groceries or clothes, using the telephone, managing money, etc.

**Self-care ADL refer to bathing, dressing and undressing, feeding self, using the toilet, taking medications, and not bedridden.

The grading of all AEs listed in the CTCAE v 5.0 will be based on the information contained therein. The grading of all other AEs, i.e., those which are not listed in the CTCAE v 5.0 will be performed by a responsible investigator, based on definitions given above.

If an AE shows an undulating course of intensity, it must be documented only once with predominant or medically most appropriate intensity / with the highest intensity grade.

- - 1. Causal Relatedness

The investigator will evaluate each AE that occurred after randomization regarding the **relatedness** with the study intervention:

| Related | There is a reasonable possibility of a causal relatedness between the event and the study intervention, i.e. the possibility for a causal relatedness between the study intervention and the AE cannot be excluded. |
| --- | --- |
| Not related | There is no reasonable possibility of a causal relatedness between the AE and the study intervention, i.e. there is a clear alternative explanation. |

- - 1. Outcome of AEs

All subjects who have reportable AEs, whether considered associated with the use of the study intervention or not, must be monitored to determine the outcome. The clinical course of the AE will be followed up until resolution or normalization of changed laboratory parameters or until it has changed to a stable condition. This also holds for ongoing AEs/SAEs of withdrawn subjects.

The **outcome** of an AE at the time of the last observation will be classified as:

| Recovered / resolved: | All signs and symptoms of an AE disappeared without any sequels at the time of the last interrogation. |
| --- | --- |
| Recovering / resolving: | The intensity of signs and symptoms has been diminishing and / or their clinical pattern has been changing up to the time of the last interrogation in a way typical for its resolution. |
| Not recovered / not resolved: | Signs and symptoms of an AE are mostly unchanged or worsened at the time of the last interrogation. |
| Recovered / resolved with sequelae: | Actual signs and symptoms of an AE disappeared but there are sequels related to the AE. |
| Fatal: | Resulting in death. If there are more than one adverse event only the adverse event leading to death (related) will be characterized as ‘fatal‘. |
| Unknown | The outcome is unknown or implausible and the information cannot be supplemented or verified. |

- - 1. Countermeasures

The term “countermeasures” refers to the specific actions taken to treat or alleviate adverse events or to avoid their sequels. Following categories will be used to categorize the countermeasures to adverse events:

| None: | No action taken. |
| --- | --- |
| Drug treatment: | Newly-prescribed medication or change in dose of a medication. |
| Others: | Other countermeasures, e.g. an operative procedure. |

##

## Period of Observation and Documentation

Adverse events (AEs) will be ascertained by the investigators using non-leading questions, noted as spontaneously reported by the subjects to the medical staff at any time during the study or observed at any of the study visit.

The observation period begins with randomization and ends with the last study visit.

AEs with a grading ≥ 2 will be documented in the subject file and in the CRF. AEs of grade 1 will not be documented in the subject file and in the CRF. If applicable, all medical diagnoses or symptoms occurring prior to the beginning of the period of observation and documentation will be recorded in the CRF as medical history.

The following general rules apply to the documentation of the AEs and SAEs: The start date of an SAE must not be earlier than that of the corresponding AE. The end date of a SAE is typically the same as that of AE. The end date of the SAE must not be later than the end date of the corresponding AE. AEs and SAEs that are ongoing at the time of death are considered not resolved or resolving.

All SAEs and their relevance for the benefit-risk assessment of the study will be evaluated continuously during the study and for the final report. All SAEs will be documented in the CRF.

# Statistical Procedures


## Definition of Study Population to be Analyzed

The primary analysis population will be the intention-to-treat (ITT) population consisting of all randomized subjects, where each subject will be analyzed within the randomized group regardless of the actual treatment.

## Analysis Variables

The primary and secondary endpoints as defined in section 2.1 and 2.2 will be analyzed.

Baseline is defined as last assessment before randomization.

## General Considerations

Further analyses will be defined in the statistical analysis plan (SAP) which has to be authorized before analysis commences by the biometrician and the sponsor. All below mentioned analyses will be described in greater detail in the SAP.

The primary analysis will be conducted when all subjects completed visit 3 of the trial. An additional analysis will take place when all subjects completed the follow up.

SAS version 9.4 or higher will be used for analyses.

## Primary Analysis

**Efficacy analysis:** In the primary analysis, the time to relapse as defined in section 2.1 will be analyzed with a mixed cox regression model with planned treatment, strata (clozapine taken Yes/No, see section 3.4.1) and BPRS at baseline as fixed effects and site as random effect in all subjects randomized (ITT). Subjects with missing information (including death) will be considered as relapses at the time point of last information. The occurrence of other intercurrent events is considered irrelevant in defining relapse (treatment policy strategy). Patients without relapse until visit 3 will be censored at visit 3 (due to possible change of intervention). The middle time point between date of awareness and the prior visit will be used to calculate the time to relapse.

The treatment effect will be tested using a two-sided alpha of 0.05 and in addition, a 95%-confidence interval will be calculated.

## Secondary Analyses

The number of relapse free subjects at the end of phase II will be analyzed by a mixed logistic regression model (two-sided alpha=0.05) with planned treatment, strata and BPRS at baseline as fixed effects and site as random effect in all subjects randomized (ITT).

BPRS, GAF, PANSS, HAMD, NCRS-dv, Q-LES-Q-18, SSMIS-SF and stigma-stress-scale will be categorized considering relapses and dropouts as additional categories. These will be tabulated using standard descriptive measures including number of non-missing values, mean, standard deviation, extrema and quartiles.

Furthermore, to analyze BPRS, GAF, PANSS, HAMD, NCRS-dv, Q-LES-Q-18, SSMIS-SF and stigma-stress-scale per time-point linear mixed models with the respective scale at baseline, strata and treatment as fixed effects and site as random effect will be used.

An explorative analysis will be carried out for all recruited subjects, where a logistic regression will be performed for the rate of inclusion in the randomized trial on baseline criteria. This way, subjects likely to profit from a first session of ECT can be determined based on clinical criteria.

**Safety analysis:** Frequencies of subjects experiencing at least one adverse event (AE) will be displayed according to MedDRA terminology. MMSE and THINC-it will be analyzed by group using standard descriptive measures.

## Interim Analyses

No interim analysis regarding efficacy or futility is planned, but two safety analyses for the DSMB.

## Sensitivity Analyses

As sensitivity analysis, the date of awareness will be used to calculate the time to relapse. Furthermore, the primary analysis is repeated using a log-rank-test.

## Subgroup Analyses

Subgroup analyses by academic/non-academic site and strata will be carried out.

## Sample Size / Power Calculation

Relapse rates after 6 months of treatment in phase II are estimated to be 90% in the TAU arm and 40% in the mECT + TAU arm based on a clinical trial with a similar study design, a systematic review and a recent open label CRS mECT study (24, 25, 42). In this calculation, more conservative relapse rates of 85% (TAU) and 45% (mECT + TAU) are assumed; although most evidence indicates even lower relapse rates for mECT + TAU, but our subjects might be more severely affected drug resistant subjects. We are aiming for sufficient power for the composite endpoint strategy where missing values are treated as relapses, which increases the relapse rates. With expected rates of missing values of 15% (TAU) and 30% (mECT + TAU) due to higher inconvenience with mECT +TAU, the relapse rates for planning the sample size are 87.25% (TAU) and 61.5% (mECT + TAU). This yields hazard rates of 0.3433 and 0.1591 and a hazard ratio of 0.4634, which in this case is a conservative approach.

Including 84 subjects (42 per group) in phase II, a difference could be found with a power of 0.85 using a cox regression with alpha= 0.05 (two-sided) and considering the reduced effect due to missing values as specified above. Even if the expected hazard rate in the MECT+TAU arm is 0.17 (≙ 63.9%) e.g. due to lower effects or due to a bias because of different observation schedules, the power is still ~80%. (The necessary sample size was simulated using R.) 140 subjects must be enrolled in phase I, if 60% of subjects are responders and willing to participate in phase II of the trial.

**Compliance / Rate of loss to follow up**

140 subjects must be enrolled, if 60% of subjects are willing to participate in phase II of the trial. We then expect withdrawal rates in phase II of 15% (TAU) and 30% (mECT + TAU) due to higher inconvenience with mECT.

Data Management

For more details refer to the corresponding data management plan (DMP). All data management activities will be conducted according to the current SOPs.


## Data Collection and Handling

In this study, REDCap (43, 44) is used as the clinical data management system for data collection by using an electronic CRF (eCRF) with remote data entry (RDE).

All protocol-required information collected during the study must be entered into the eCRF by the investigator or a designated representative. There must not be subject identifying data in the eCRF. There must be no data that are inconsistent between eCRF and source documents. In addition, source documents must reflect that the subject has been enrolled in this study and must include all medical information necessary for appropriate medical care outside of the study. Data corrections must always be justified. Data entry should be completed within 7 days after the respective study procedure. Any pending data entries have to be completed immediately after the final examination. Missing data should be explained. Completeness and correctness of all data entries in the eCRF have to be confirmed by dated electronic signatures of the responsible investigator.

## Data Coding

Coding of adverse events will be done according to the MedDRA coding systems.

## Data Cleaning and Quality Checks

Data entries will be checked for plausibility and consistency. The checks are defined in the study specific data validation plan (DVP). In case of implausibilities, 'warnings' will be generated during data entry (edit checks). The responsible investigator or a designated representative must then either correct the entered data or confirm its correctness by giving an explanation. The responsible data manager will check all explanations and resolves the warnings if the explanation is appropriate. The responsible monitor may raise electronic questions (monitor queries) to the responsible investigator as well. The investigator or a designated representative should answer queries within one week. The responsible monitor will verify the answer and resolve the query if the answer is appropriate. A similar query flow can be used by the data manager (DM query).

All missing data or inconsistencies have to be clarified by the responsible investigator prior to database lock. If no further corrections in the database are required it will be declared as locked and used for statistical analysis.

# Archiving and Storage


The coordinating investigator and investigator(s) will archive the Trial Master File (TMF) / Investigator Site File (ISF) as well as source data for at least 10 years after the end of the study.

Any change of data ownership shall be documented. All data shall be made available if requested by relevant authorities.

# Regulatory, Ethical and Study Oversight Considerations


## Compliance Statement

This study will be conducted in compliance with the protocol and in accordance with the following regulatory requirements:

- Consensus ethical principles derived from international guidelines including the Declaration of Helsinki and Council for International Organizations of Medical Sciences (CIOMS) International Ethical Guidelines,
- Applicable ICH GCP Guidelines,
- Applicable laws and regulations.

## Data Protection and Subject Privacy

Data obtained during the study will be handled pursuant to the EU General Data Protection Regulation (GDPR) and national regulatory requirements.

To ensure confidentiality of records and personal data, only pseudonymized data will be transferred to coordinating investigator by using a subject identification number instead of the subject’s name. The code is only available at the site and must not be forwarded to coordinating investigator. Access to the subject’s files and clinical data is strictly limited. Personalized treatment data may be given to the subject’s personal physician or to other appropriate medical personnel responsible for the subject’s welfare. Study specific data generated at the site need to be available for inspection on request by the participating investigators, coordinating investigator and by the IEC.

Video recordings can only be completely pseudonymized with great effort. This pseudonymization cannot be guaranteed during the recordings in this study. Therefore, there is a very low probability that a person involved in the data analysis will recognize the participant on the recordings. For this reason, all persons involved in the analysis are subject to an absolute duty of confidentiality and may not disclose confidential information to third parties under any circumstances. The video material is encrypted and transported to the study center for data backup. In addition, the encrypted data will be sent to the respective evaluator/supervisor. The video recordings are stored in the study center on a password-protected computer that is separate from the Internet. The video material is used exclusively for research and supervision purposes. For the evaluation, the video recordings are edited in such a way that it is not possible for the blinded raters to assign them to a treatment arm.

## Approval of the Study

This study will be initiated only after all required legal documentation has been reviewed and approved by the responsible IEC according to national regulations. The same applies for the implementation of changes introduced through modifications.

## Subject Information and Informed Consent

Before being enrolled in the study, the subject must consent to participate after being fully informed by the investigator or a designated member of the investigating team about the nature, importance, risks and individual consequences of the study and his/her right, to terminate participation at any time. The subject should also have the opportunity to consult the investigator about the details of the study. The investigator shall emphasize that subjects are completely free to refuse participation or to withdraw later on at any time, without suffering consequences for future care and without the need to justify.

Each subject will be informed that his/her source records may be reviewed by the study monitor/ quality assurance auditor and that the video recordings will be reviewed by an independent evaluator/supervisor. These persons are bound by confidentiality obligations.

After reading the informed consent document, subject and investigator conducting the informed consent discussion must sign and personally date the informed consent form. A copy of the signed informed consent document must be given to the subject; the original will be filed in the ISF by the investigator. The consent process must be documented in the patient file.

Written subject information must be in a language understandable to the subject and must specify who informed the subject.

The subjects will be informed as soon as possible if new information may influence his/her decision to participate in the study. The communication of this information should be documented.

The above-named general provision concerning information and obtaining informed consent apply likewise to the legally designated representative.

## Data Safety Monitoring Board (DSMB)

Although safety monitoring is an essential and integral part of any study, a DSMB is typically indicated for studies that aim to save lives, monitor safety in long-term studies including non-life-threatening diseases, or reduce the risk of serious health consequences. Therefore, there is no typical DSMB indication for the MECT Resist study. However, due to the long inclusion period and the large number of participating centers, a DSMB will be established to ensure integrity and validity of the data. ECT is a medically safe procedure, but this fact is sometimes not well publicized, which is another (for the public relevant) reason for implementing a DSMB.

The DSMB consists of three independent psychiatrists who are not involved in the conduct of the study and will meet twice during the course of the study to evaluate the tolerability of the treatment.

# Quality Control and Quality Assurance


## Quality Assurance

A risk-based approach is used for study quality management. It is initiated by the assessment of critical data and processes for subject protection and reliability of the results as well as identification and assessment of associated risks. The rationale and strategies for risk management during study conduct including monitoring approaches and other processes focusing on areas of greatest risk will be documented.

A quality assurance audit of this study may be conducted at any time during or after completion of the study. Representatives of the coordinating investigator may visit the site to audit compliance with applicable regulatory requirements and the coordinating investigator’s policies. Audits will require access to all study records and source documents. The investigator and site personnel must be available for consultation during site audits.

## Monitoring

Following a risk-based approach, a combination of monitoring techniques (remote or on-site visits) will be used to monitor the study.

As the monitoring strategy will consider current aspects of risk-based quality management, frequency of monitoring activities per site will vary depending on recruitment and general performance, e.g. quality of documentation.

The monitor will ensure that the study is conducted according to the protocol and applicable regulatory requirements by reviewing essential records, source documents and entries into the CRFs. The monitor will document the visits in a report for the coordinating investigator. The site will be provided with a follow-up letter about the findings and the necessary actions to be taken.

In addition to on-site monitoring visits, sites may be contacted remotely. It is expected that during these remote contacts, site personnel will be available to provide an update on the progress of the study at the site. Details of monitoring will be defined in the monitoring plan.

In case of critical findings during monitoring or an audit, the site might be closed prematurely by the coordinating investigator.

According to ICH-GCP the investigator(s) / institution(s) must provide direct access to source data / documents for study related monitoring, audits and inspections by regulatory authorities. Via the written informed consent each subject has agreed to grant study related monitors, auditors and inspectors from regulatory authorities direct access to his/her original medical records.

In case of electronic medical records, the monitor’s / auditor’s access must be restricted to the subjects. If this is not possible the files have to be reviewed in the presence of site staff. The electronic medical record should have an accessible audit trail.

# Administrative Agreements


## Financing of the Study

The study will be financed using funds of the German Federal Ministry of Education and Research (project number: 01KG2401).

## Publication Policy

A clinical study report will be written after all subjects have completed the study.

The rights of the investigator and coordinating investigator regarding publication of the study results are described in the investigator contract. In general, no study results should be published prior to finalization of the clinical study report.

# References

1. Insel TR, Scolnick EM. Cure therapeutics and strategic prevention: raising the bar for mental health research. Mol Psychiatry. 2006;11(1):11-7.
2. Robinson DG, Woerner MG, McMeniman M, Mendelowitz A, Bilder RM. Symptomatic and functional recovery from a first episode of schizophrenia or schizoaffective disorder. Am J Psychiatry. 2004;161(3):473-9.
3. Harrison G, Hopper K, Craig T, Laska E, Siegel C, Wanderling J, et al. Recovery from psychotic illness: a 15- and 25-year international follow-up study. Br J Psychiatry. 2001;178:506-17.
4. Marwaha S, Johnson S, Bebbington P, Stafford M, Angermeyer MC, Brugha T, et al. Rates and correlates of employment in people with schizophrenia in the UK, France and Germany. Br J Psychiatry. 2007;191:30-7.
5. Gustavsson A, Svensson M, Jacobi F, Allgulander C, Alonso J, Beghi E, et al. Cost of disorders of the brain in Europe 2010. Eur Neuropsychopharmacol. 2011;21(10):718-79.
6. Insel TR. Rethinking schizophrenia. Nature. 2010;468(7321):187-93.
7. Walker ER, McGee RE, Druss BG. Mortality in mental disorders and global disease burden implications: a systematic review and meta-analysis. JAMA Psychiatry. 2015;72(4):334-41.
8. Zink M, Englisch S, Meyer-Lindenberg A. [Polypharmacy in schizophrenia]. Nervenarzt. 2011;82(7):853-8.
9. Cipriani A, Boso M, Barbui C. Clozapine combined with different antipsychotic drugs for treatment resistant schizophrenia. Cochrane Database Syst Rev. 2009(3):CD006324.
10. Barber S, Olotu U, Corsi M, Cipriani A. Clozapine combined with different antipsychotic drugs for treatmentresistant schizophrenia. Cochrane Database Syst Rev. 2017;3:CD006324. 19. DGPPN. Indikationen zur EKT, Nervenheilkunde. 2022;41,706-709
11. DGPPN. S3-Leitlinie Schizophrenie. Langfassung Version 1.0, 2019.
12. DGPPN. Indikationen zur EKT, Nervenheilkunde. 2022;41,706-709
13. Biedermann F, Pfaffenberger N, Baumgartner S, Kemmler G, Fleischhacker WW, Hofer A. Combined clozapine and electroconvulsive therapy in clozapine-resistant schizophrenia: clinical and cognitive outcomes. J ECT. 2011;27(4):e61-2.
14. Taylor P, Fleminger JJ. ECT for schizophrenia. Lancet. 1980;1(8183):1380-2.
15. Kristensen D, Bauer J, Hageman I, Jorgensen MB. Electroconvulsive therapy for treating schizophrenia: a chart review of patients from two catchment areas. Eur Arch Psychiatry Clin Neurosci. 2011;261(6):425-32.
16. Abraham KR, Kulhara P. The efficacy of electroconvulsive therapy in the treatment of schizophrenia. A comparative study. Br J Psychiatry. 1987;151:152-5.
17. Tang WK, Ungvari GS. Efficacy of electroconvulsive therapy in treatment-resistant schizophrenia: a prospective open trial. Prog Neuropsychopharmacol Biol Psychiatry. 2003;27(3):373-9.
18. Tharyan P, Adams CE. Electroconvulsive therapy for schizophrenia. Cochrane Database Syst Rev. 2005(2):CD000076.
19. Grover S, Chakrabarti S, Hazari N, Avasthi A. Effectiveness of electroconvulsive therapy in patients with treatment resistant schizophrenia: A retrospective study. Psychiatry Res. 2017;249:349-53.
20. Petrides G, Malur C, Braga RJ, Bailine SH, Schooler NR, Malhotra AK, et al. Electroconvulsive therapy augmentation in clozapine-resistant schizophrenia: a prospective, randomized study. Am J Psychiatry. 2015;172(1):52-8.
21. Masoudzadeh A, Khalilian AR. Comparative study of clozapine, electroshock and the combination of ECT with clozapine in treatment-resistant schizophrenic patients. Pak J Biol Sci. 2007;10(23):4287-90.
22. Sanghani SN, Petrides G, Kellner CH. Electroconvulsive therapy (ECT) in schizophrenia: a review of recent literature. Curr Opin Psychiatry. 2018;31(3):213-22.
23. Kim HS, Kim SH, Lee NY, Youn T, Lee JH, Chung S, et al. Effectiveness of Electroconvulsive Therapy Augmentation on Clozapine-Resistant Schizophrenia. Psychiatry Investig. 2017;14(1):58-62.
24. Ward HB, Szabo ST, Rakesh G. Maintenance ECT in schizophrenia: A systematic review. Psychiatry Res. 2018;264:131-42.
25. Braga RJ, John M, Schooler NR, Bailine SH, Malur C, Mendelowitz A, et al. Continuation Electroconvulsive Therapy for Patients With Clozapine-Resistant Schizophrenia: A Pilot Study. J ECT. 2019;35(3):156-60.
26. Levy-Rueff M, Gourevitch R, Loo H, Olie JP, Amado I. Maintenance electroconvulsive therapy: an alternative treatment for refractory schizophrenia and schizoaffective disorders. Psychiatry Res. 2010;175(3):280-3.
27. Espinoza RT, Kellner CH. Electroconvulsive Therapy. N Engl J Med. 2022;386(7):667-72.
28. Dalby M, Annas P, andMe Research T, Harrison JE. Further validation of the THINC-it tool and extension of the normative data set in a study of n = 10.019 typical controls. Int J Methods Psychiatr Res. 2022;31(4):e1922.
29. McIntyre RS, Subramaniapillai M, Park C, Zuckerman H, Cao B, Lee Y, et al. The THINC-it Tool for Cognitive Assessment and Measurement in Major Depressive Disorder: Sensitivity to Change. Front Psychiatry. 2020;11:546.
30. Szmyd JK, Lewczuk K, Teopiz KM, McIntyre RS, Wichniak A. THINC-Integrated Tool (THINC-it): A Brief Measurement of Changes in Cognitive Functioning and Its Correlation with the Life Quality of Patients with Schizophrenia and Related Disorders-A Pilot Study. Brain Sci. 2023;13(3).
31. Semkovska M, McLoughlin DM. Objective cognitive performance associated with electroconvulsive therapy for depression: a systematic review and meta-analysis. Biol Psychiatry. 2010;68(6):568-77.
32. Yoldi-Negrete M, Gill LN, Olivares S, Lauziere A, Desilets M, Tourjman SV. The effect of continuation and maintenance electroconvulsive therapy on cognition: A systematic review of the literature and meta-analysis. J Affect Disord. 2022;316:148-60.
33. Overall JEG, D.R. The brief psychiatric rating scale. Psychological Reports. 1962;10:799-812.
34. Aas IH. Guidelines for rating Global Assessment of Functioning (GAF). Ann Gen Psychiatry. 2011;10:2.
35. Kay SR, Fiszbein A, Opler LA. The positive and negative syndrome scale (PANSS) for schizophrenia. Schizophr Bull. 1987;13(2):261-76.
36. Hamilton M. A rating scale for depression. J Neurol Neurosurg Psychiatry. 1960;23:56-62.
37. Hirjak D, Thomann PA, Northoff G, Kubera KM, Wolf RC. [German version of the Northoff catatonia rating scale (NCRS-dv) : A validated instrument for measuring catatonic symptoms]. Nervenarzt. 2017;88(7):787-96.
38. Ritsner M, Kurs R, Gibel A, Ratner Y, Endicott J. Validity of an abbreviated quality of life enjoyment and satisfaction questionnaire (Q-LES-Q-18) for schizophrenia, schizoaffective, and mood disorder patients. Qual Life Res. 2005;14(7):1693-703.
39. Corrigan PW, Michaels PJ, Vega E, Gause M, Watson AC, Rusch N. Self-stigma of mental illness scale--short form: reliability and validity. Psychiatry Res. 2012;199(1):65-9.
40. Rusch N, Corrigan PW, Wassel A, Michaels P, Olschewski M, Wilkniss S, et al. A stress-coping model of mental illness stigma: I. Predictors of cognitive stress appraisal. Schizophr Res. 2009;110(1-3):59-64.
41. Folstein MF, Folstein SE, McHugh PR. "Mini-mental state". A practical method for grading the cognitive state of patients for the clinician. J Psychiatr Res. 1975;12(3):189-98.
42. Chanpattana W, Chakrabhand ML, Sackeim HA, Kitaroonchai W, Kongsakon R, Techakasem P, et al. Continuation ECT in treatment-resistant schizophrenia: a controlled study. J ECT. 1999;15(3):178-92.
43. Harris PA, Taylor R, Thielke R, Payne J, Gonzalez N, Conde JG. Research electronic data capture (REDCap) – A metadata-driven methodology and workflow process for providing translational research informatics support, J Biomed Inform. 2009;42(2):377-81.
44. Harris PA, Taylor R, Minor BL, Elliott V, Fernandez M, O’Neal L, McLeod L, Delacqua G, Delacqua F, Kirby J, Duda SN, REDCap Consortium, The REDCap consortium: Building an international community of software partners, J Biomed Inform. 2019 May 9 [doi: 10.1016/j.jbi.2019.103208].
